# Light-Triggered Reversible Change in the Electronic Structure of MoO_3_ Nanosheets via an Excited-State Proton Transfer Mechanism

**DOI:** 10.1021/acs.nanolett.3c04209

**Published:** 2024-01-30

**Authors:** Yuval Gilad Barzilay, Anna Yucknovsky, Nadav Amdursky

**Affiliations:** Schulich Faculty of Chemistry, Technion - Israel Institute of Technology, Haifa 3200003, Israel

**Keywords:** pyranine, nanosheets, plasmon resonance, molybdenum oxide

## Abstract

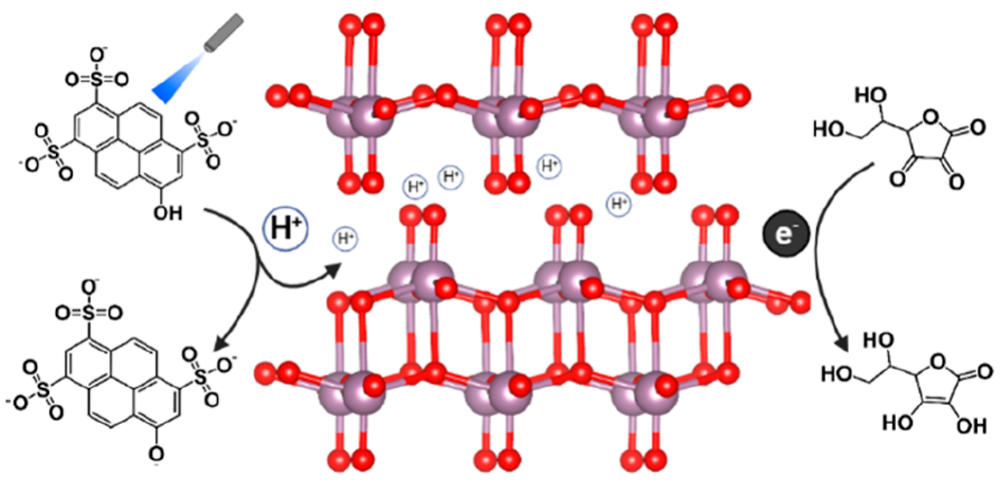

Light is an attractive source of energy for regulating stimulus-responsive chemical systems. Here, we use light as a gating source to control the redox state, the localized surface plasmonic resonance (LSPR) peak, and the structure of molybdenum oxide (MoO_3_) nanosheets, which are important for various applications. However, the light excitation is not that of the MoO_3_ nanosheets but rather that of pyranine (HPTS) photoacids, which in turn undergo an excited-state proton transfer (ESPT) process. We show that the ESPT process from HPTS to the nanosheets and the intercalation of protons within the MoO_3_ nanosheets trigger the reduction of the nanosheets and the broadening of the LSPR peak, a process that is reversible, meaning that in the absence of light, the LSPR peak diminishes and the nanosheets return to their oxidized form. We further show that this reversible process is accompanied by a change in the nanosheet size and morphology.

Molybdenum oxide (MoO_3_) is a versatile transition metal oxide with numerous applications in different fields, such as catalysis,^1^ biology,^2^ sensors,^3^ and batteries.^4^ This versatility arises from its structural diversity and variety in stoichiometry.^5^ MoO_3_ structures can be described as a planar layer of double-stacked octahedral MoO_6_, in which the atoms are strongly bound to one another. The connection between two octahedral blocks makes use of a bridging oxygen atom shared between two Mo atoms.^6^ The layers are vertically connected by weak van der Waals forces formed by terminal oxygens, thus creating an array of nanosheets (Figure 1a, inset). This layered structure of MoO_3_ is not hindered by the occurrence of nonstoichiometric events such as oxygen vacancies and reduced Mo atoms (i.e., Mo^5+^ and Mo^4+^) in the lattice, and the intercalation of small ions such as H^+^ and Li^+^ between layers in the van der Waals gap does not cause major deviations in the crystal structure.^5,6^ In the event of intercalation of H^+^ into the van der Waals gap, it can interact with an oxygen atom, affecting the electronic structure of MoO_3_.^7^ This can lead to the reduction of the Mo^6+^ atoms to Mo^5+^ in the presence of various reducing agents, such as NaBH_4_,^8^ ascorbic acid,^9^ and glutathione.^10^ The conversion of Mo^6+^ to Mo^5+^ partially fills the 4d orbital of the Mo atom and generates an oxygen vacancy, which increases the number of delocalized electrons within the crystal. The nonstoichiometric reduction of the MoO_3_ nanosheets to MoO_3–*x*_ is accompanied by a change in the color of the nanosheets from white to blue, resulting from a localized surface plasmon resonance (LSPR), i.e., the oscillation of the conduction band electrons at a frequency equal to that of the incident light.^5,11−13^ This LSPR can occur only if the d orbitals are filled with electrons, thus creating a delocalized “d band”.^14,15^

**Figure 1:**
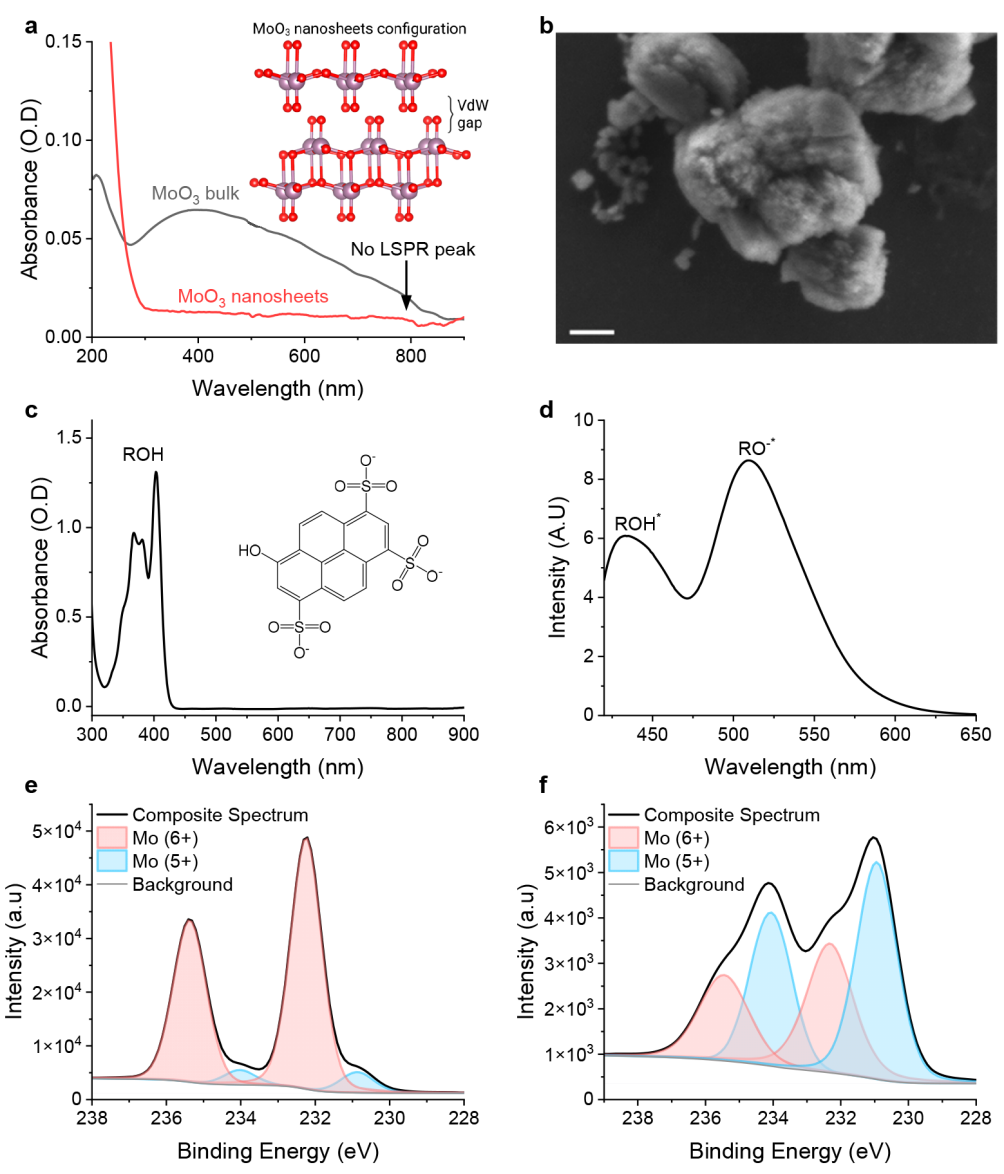
(a) UV–vis absorption spectrum of bulk MoO_3_ compared to the nanosheet configuration. The inset shows a schematic crystal structure of the MoO_3_ nanosheet configuration. (b) SEM image of MoO_3_ nanosheets. The scale bar represents 100 nm. (c and d) UV–vis absorption and fluorescence (λ_ex_ = 400 nm) measurements, respectively, of HPTS in the solution used in this study (50% ethanol). The ROH/ROH*/RO^–^* bands are indicated. The inset in panel c shows a molecular scheme of HPTS. (e and f) XPS spectra of MoO_3_ before and after the addition of ascorbic acid, respectively.

In this study, we aim to modulate the electronic structure of MoO_3–*x*_ nanosheets related to their redox state and the formation of an LSPR. Because the conversion of MoO_3_ to MoO_3–*x*_ can be achieved under acidic conditions in the presence of a reducing agent,^9,10^ the intensity of the LSPR peak increases with acidity, i.e., the concentration of protons in solution.^10^ Here, we introduce a new mechanism for the reduction of MoO_3_ via an excited-state proton transfer (ESPT) mechanism from a photoacid donor. In this mechanism, we induce a direct or mediated proton transfer from the photoacid to the MoO_3–*x*_ nanosheets without the acidification of the solvent. This type of photoacid is an -OH aryl molecule (ROH) that can serve as a proton donor, i.e., dissociate, only upon light excitation: . The driving force for the proton dissociation is the fundamentally different p*K*_a_ values between the ground and excited states of the photoacid. For the common 8-hydroxypyrene-1,3,6-trisulfonate (HPTS) photoacid that we used in this study, the ground- and excited-state p*K*_a_ values are 7.4 and 0.4, respectively. Importantly, the proton dissociation of such photoacids is reversible, and upon returning to the ground state, the deprotonated species (RO^–^) is reprotonated.^16−19^ The reversible ESPT from a photoacid to a nearby proton acceptor can be utilized to light-trigger various processes that are dependent on protonation,^20−26^ which are conceptually acid–base reactions. Here, we show that MoO_3_ nanosheets can serve as proton acceptors to a photoacid, resulting in a light-triggered change in the redox state of MoO_3_ nanosheets to nonstoichiometric MoO_3–*x*_ nanosheets and reversible oxidation in the dark. This is also the main difference between using a regular acid and a photoacid. The use of an acid is not reversible, and there is a need to add a base to induce its reversibility; the use of a photoacid is reversible, i.e., in the dark the driving force is toward reprotonation. It is also important to note that due to the immediate reprotonation of RO^–^ in the ground state in an aqueous solution, the use of such photoacids does not result in a stable pH jump of the solution,^27^ which is different from the case for other types of photoacids, such as the merocyanine–spiropyran system that can result in a stable pH jump.^28^ Hence, to use the HPTS photoacid to control pH-dependent processes, the terminal proton acceptor has to be the system or molecule in question.

The MoO_3_ nanosheet solution was prepared via a liquid exfoliation methodology.^29,30^ MoO_3_ powder was ground and dispersed in a H_2_O/ethanol (1:1) solution, followed by sonication of the solution and extraction (further details in the experimental section of the Supporting Information). In line with previous studies, the optical absorption of the formed nanosheets did not exhibit a broad absorption throughout the visible range, which is different from the absorption of bulk MoO_3_ (Figure 1a).^31^ Scanning electron microscopy (SEM) characterization revealed that the nanosheets were organized in three-dimensional (3D) structures of “layered nanosheets”, i.e., singular nanosheets organized parallel to one another, forming a block (Figure 1b). These blocks are aggregated together in different orientations from each other but do not cross each other.

In our work, and in line with previous studies,^9,10^ we hypothesize that the ESPT from the photoacid to the nanosheets can result in a rapid change in the electronic structure of the reduced material, i.e., MoO_3–*x*_. Accordingly, we must first confirm that under the solvent conditions used here (50% ethanol), the photoacid [HPTS (Figure 1c, inset)] is in its ROH state and that upon excitation (ROH*), it is deprotonated to its RO^–*^ state; thus, it can serve as a proton donor. Indeed, ultraviolet–visible (UV–vis) absorption shows that the photoacid is at its ROH state in its ground state (Figure 1c), and fluorescence measurement shows a clear RO^–^* peak upon excitation of the ROH (at 405 nm) (Figure 1d), thus indicating an ESPT process, i.e., the deprotonation of HPTS in the excited state. It is important to note here that many external cues can result in the reduction of the MoO_3_ nanosheets, such as UV light^32,33^ or even the discussed sonication process.^29^ Accordingly, the starting material in our work already consists of partially reduced MoO_3–*x*_ nanosheets, as otherwise we could not have decoupled the ESPT process as the cause of the reduction to any light-induced initial reduction of the nanosheets (discussed and elaborated below). To form such partially reduced MoO_3–*x*_ nanosheets, we used ascorbic acid as a common reducing agent for MoO_3_,^9,10^ resulting in a clear change in the color of the nanosheet solution from a colorless solution to a light blue solution. Unlike the original solution of the MoO_3_ nanosheets before the addition of ascorbic acid, the solution of the reduced MoO_3–*x*_ nanosheets exhibited an LSPR peak in the NIR region (Figure S1).

We further followed the reduction process by X-ray photoelectron spectroscopy (XPS) measurements. There are four peaks in the XPS spectrum of the nanosheets associated with Mo 3d: 3d_5/2_ and 3d_3/2_ peaks of Mo in the 6+ oxidation state at 232.2 and 235.4 eV, respectively, and 3d_5/2_ and 3d_3/2_ peaks of Mo in the 5+ oxidation state at 230.9 and 234.0 eV, respectively.^9,34^ Before the addition of ascorbic acid (Figure 1e), the Mo^6+^:Mo^5+^ ratio is 92:8, indicating low levels of reduced Mo that could be a result of the sonication process during synthesis. However, as discussed, the presence of a minute amount of Mo^5+^ in the solution did not exhibit an LSPR peak (Figure 1a). After the addition of ascorbic acid (Figure 1f), the Mo^6+^:Mo^5+^ ratio changed to 42:58. The vast increase in the amount of Mo^5+^ correlates with the result that ascorbic acid induces the formation of the LSPR peak.

As an important sanity check to our hypothesis here, we followed the intensity and wavelength of the LSPR peak as a function of the acidity of its environment. We observed that the more acidic the solution (by adding HCl), the higher the absorbance of the LSPR peak, and vice versa, the more basic the solution (by adding NaOH), the lower the absorbance of the peak (Figure S1). We also observed a change in the wavelength maximum location; in the acidic solution, the maximum appeared at shorter wavelengths, and in the basic solution, the maximum appeared at longer wavelengths (Figure S1).

The next step is to examine whether ESPT from the photoacid can manipulate the electronic structure of the reduced MoO_3–*x*_ nanosheets. We exposed the system in the presence of HPTS to a 405 nm light source and followed changes in the NIR region. Unexpectedly, we found an irreversible increase in the LSPR peak following the first exposure of the system to light (Figure S2), meaning that upon turning off the light the intensity of the LSPR peak did not decrease to its initial value. Unlike the change in the intensity of the LSPR peak observed in the first cycle of irradiation, upon further exposure of the system to light, we observed a hypsochromic broadening with no changes to the LSPR peak maxima intensity, i.e., an increase only on the lower-wavelength side of the LSPR peak (Figure 2a). Importantly, and unlike the irreversible change of the first exposure, the observed hypsochromic broadening was reversible, and when the light was turned off, it returned to its initial shape. This change and its reversibility can be cycled for several exposures to light (Figure 2a). By following the increase in the blue edge of the LSPR peak as a function of irradiation and dark times (Figure 2b), we can extract the kinetics of the process. In the transients shown in Figure 2b, we can see that the change in the LSPR peak intensity occurred immediately after the excitation of HPTS. Nevertheless, it is important to decouple between the ultrafast ESPT process that is on the (sub)nanosecond time scale and the changes in the redox state of the nanosheets that are on the minute time scale and result from numerous ESPT processes. From a kinetic point of view, the increase in the absorption following excitation can be easily fitted to a single-exponential association with a time constant of 0.58 min^–1^. However, the decrease in the absorption when the light is turned off is more complicated, showing a two-step process composed of a fast initial process followed by a slow nonlinear process (*vide infra*).

**Figure 2:**
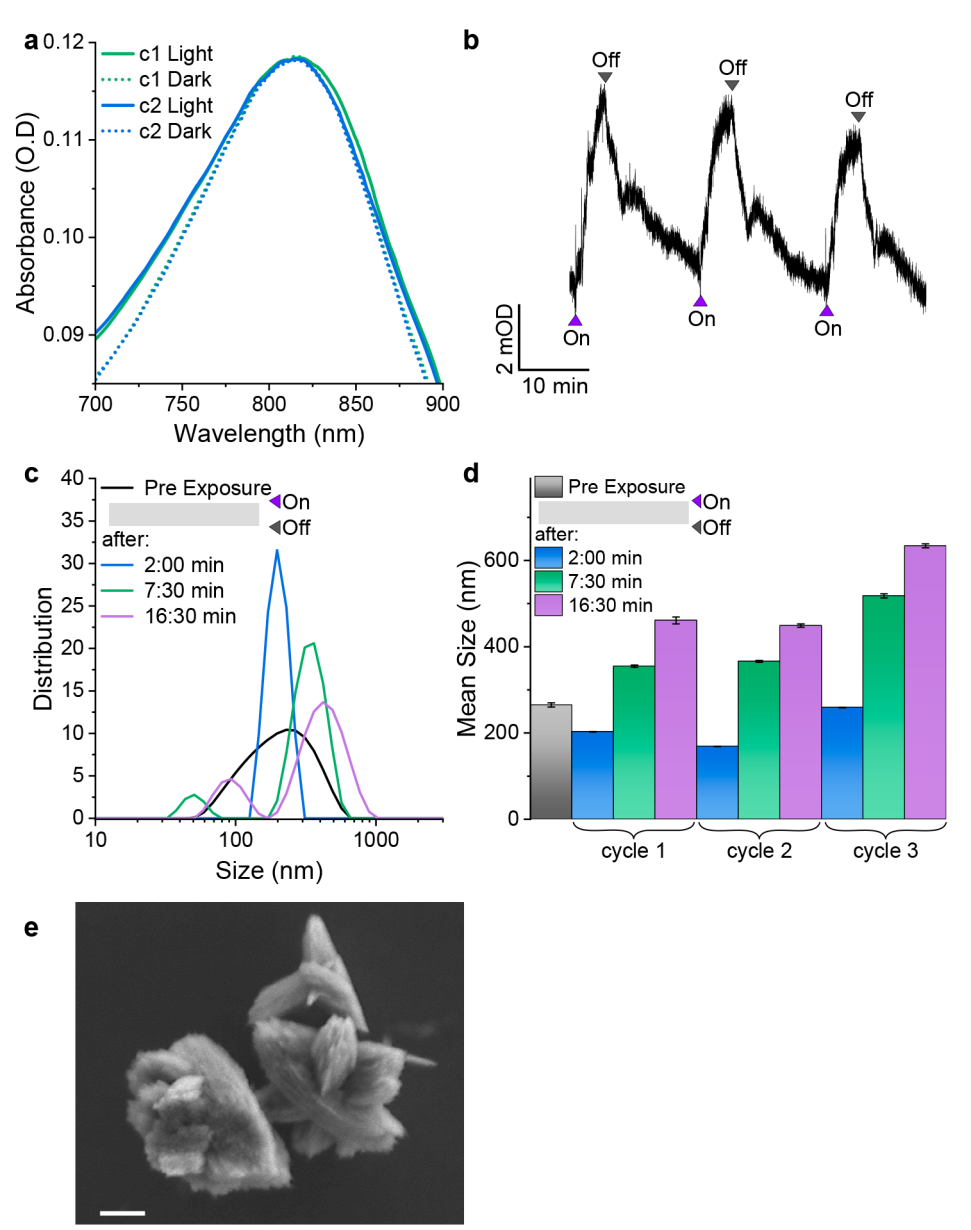
(a) Changes in the UV–vis absorption of the LSPR peak upon two cycles (c): light, immediately after 405 nm exposure for 5 min; dark, after 30 min in the dark. (b) Transient of the UV–vis absorption at 750 nm upon three cycles of light (405 nm) and dark. (c) DLS measurements of the MoO_3_ nanosheets in solution before exposure (405 nm for 5 min) and after exposure at different time points in the dark. (d) Mean hydrodynamic size of the nanosheets at the mentioned time points at three consecutive cycles. (e) SEM of the MoO_3_ nanosheets after 405 nm exposure for 5 min showing an interlaced configuration. The scale bar represents 100 nm.

To validate that the ESPT process is the governing mechanism in the observed changes in the LSPR peak of the reduced MoO_3–*x*_ nanosheets upon irradiation, we performed two important control experiments. The first control was replacing the HPTS with a methylated version of it, 8-methoxypyrene-1,3,6-trisulfonate (MPTS). MPTS has a structure and optoelectronic properties similar to those of HPTS, but it cannot deprotonate; hence, it cannot serve as a proton donor.^35,36^ The second control experiment was to remove HPTS from the system altogether, which aimed to decipher any artifacts that might arise from light–matter interactions of our light source with the MoO_3–*x*_ nanosheets themselves. Importantly, both control experiments show that the observed changes in the LSPR peak can be ascribed only to the presence of HPTS and to the ESPT from it, and no changes were observed without having it in the system or replacing it with MPTS (Figure S3).

In the next step, we used dynamic light scattering (DLS) measurements to observe any change in the physical properties of the MoO_3–*x*_ nanosheets, their hydrodynamic size, following the ESPT process from HPTS to the nanosheets, and their reversibility (unlike before, DLS measurements could not be performed during illumination). The DLS measurements show an interesting nonlinear trend in the size of the nanosheets following the excitation by HPTS (Figure 2c). In the first minutes after the light had been turned off, we observed a decrease in the particle size compared to their initial preillumination state. However, with time in the dark, we observed an increase in the particle size, where they reached much larger sizes compared to that of the initial state. Importantly, we found that the change in the hydrodynamic size of the nanosheets is reversible, meaning that re-exciting the sample resulted in a large decrease in the particle size followed by their slow growth (Figure 2d). Contrary to the results of the UV–vis optical measurements, in which the first cycle exhibited an irreversible trend, the nanosheet size displayed a reversible trend from the first cycle.

To further investigate any change in the morphology and organization of the nanosheets following the ESPT process from the photoacid, we followed the process using SEM characterization. As discussed above, the nanosheets prior to light exposure are organized in a layered manner (Figure 1b). We found that after exposure to light in the presence of HPTS, the nanosheets display a different type of spatial organization, which can be termed “interlaced nanosheets” (Figure 2e). These interlaced nanosheets still consist of “nanosheet blocks”, yet the “nanosheet blocks” intersect with each other as opposed to the “layered nanosheets”, which are merely organized in different orientations to one another. Hence, we can suggest at this stage that the ESPT process from the excited photoacid to the MoO_3–*x*_ nanosheets results in some breakage of the structure, supposedly even breaking the “nanosheet blocks”. Accordingly, after light exposure, the nanosheets aggregate into a new less organized structure.

The main remaining question is the mechanism by which an ESPT process from the photoacid to reduced MoO_3–*x*_ nanosheets can alter their electronic structure and physical structure. As discussed, the initial state of our system consists of partially reduced MoO_3–*x*_ nanosheets that were obtained by using ascorbic acid as a reducing agent. Ascorbic acid can irreversibly donate its electrons to the nanosheets, resulting in their reduction and intercalation of protons (from the solution) to the exposed oxygen atoms within the van der Waals gaps of MoO_3_.^7,8^ After donating its electrons, ascorbic acid becomes dehydroascorbic acid and thus cannot further participate in the process.^37^ The reduction process is accompanied by the release of water molecules from the lattice. As the oxygen is released from the lattice structure, a Mo–O bond breaks, leaving an oxygen vacancy neighbored by reduced Mo^6+^ cations to Mo^5+^, thus allowing electrons to fill an empty 4d orbital of the Mo cations, resulting in an LSPR peak.^9^

The described equilibrated system is the starting point of our system, i.e., before the irradiation of HPTS (step 0 in Scheme 1a). Following the irradiation of HPTS, we should distinguish between two dynamic processes. The first is a one-step reaction occurring during irradiation, and the second is a two-step reaction occurring in the dark. Under irradiation, HPTS undergoes an ESPT process. While most of the released protons will be accepted by water in the system, followed by their recombination back with the deprotonated HPTS (RO^–^) when it returns to the ground state, some of the released protons will be accepted by the nanosheets, resulting in their intercalation in the van der Waals gap of the nanosheets (step 1 in Scheme 1a and step I in Scheme 1b). As before, the ascorbic acid present in the system can irreversibly donate the needed electrons to the intercalated protons (step 2 in Scheme 1a and step II in Scheme 1b). Accordingly, during excitation, protons accumulate in the van der Waals gap and are constantly being activated by ascorbic acid, accompanied by water formation and a metastable oxygen vacancy within the lattice structure (step III in Scheme 1b). The Mo cations adjacent to the oxygen vacancy are reduced from Mo^6+^ to Mo^5+^, resulting in a hypsochromic increase in the LSPR peak. From our DLS measurements, it is clear that exposure to light caused an immediate decrease in the nanosheet size. This size decrease can be attributed to the dispersion of the nanosheets in the solution and to their breaking. Comparatively, MoO_3_ is more hydrophobic than MoO_3–*x*_ due to the presence of oxygen vacancies; hence, water can interact more with the reduced nanosheets. Our findings are in line with a previous study suggesting that an increased number of oxygen vacancies in MoO_3_ nanosheets cause cracking and a reduction in the size of the nanosheets.^33^ We further followed the ζ potential of the MoO_3_ nanosheets during the reduction process and observed no change in charge (Figure S4), thus suggesting no change in the surface electrostatic in the process; i.e., the structure conserves their charge neutrality. Our results are in line with previous studies showing no net change in charge during the reduction of MoO_3_ nanosheets.^38^

**Scheme 1:**
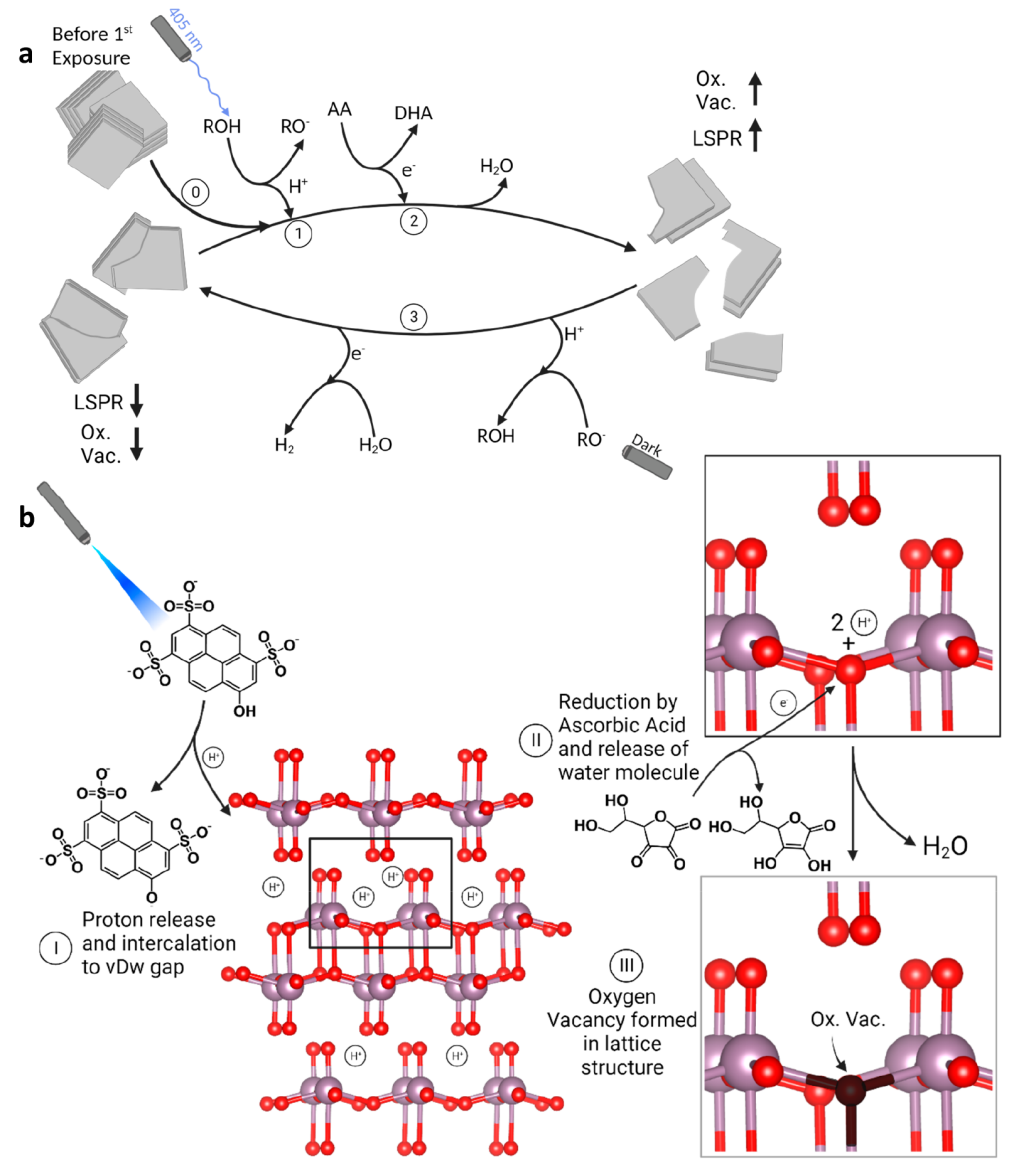
Light-Gating Mechanism of the HPTS–MoO_3–*x*_ Nanosheet System Presented (a) Schematically and (b) with the Crystal Structure of MoO_3_ (0) Reduced layered nanosheets are present in the solution before any light exposure event. (1 and I) During 405 nm illumination, the photoacid deprotonates and undergoes ESPT, where protons intercalate into the van der Waals gap within the nanosheets. (2 and II) Ascorbic acid donates its electrons, resulting in water formation and (III) oxygen vacancies in the lattice. The nanosheets undergo breaking and are more dispersed in solution. (3) In the dark, there is no ESPT, and self-healing of the oxygen vacancies occurs due to water splitting by the nanosheets. This is followed by the aggregation of nanosheets into an interlaced structure.

After the light is turned off, the reduction process is discontinued, and a two-step oxidation and aggregation process of MoO_3–*x*_ begins. Because the observed LSPR feature that was rising under irradiation starts to decrease over time in the dark, it is reasonable to claim that the oxygen vacancies are recapturing an oxygen atom in the lattice structure, which can be explained by a spontaneous self-healing process (step 3 in Scheme 1a). Initially, water molecules are absorbed at the surface vacancy sites, which is followed by electron transfer from Mo^5+^ to the oxygen atom, leading to a new Mo–O bond at the oxygen vacancy site. As a result, the formation of a new bond leads to water splitting, whereas hydroxide is incorporated into the lattice structure and protons can bind to other surface oxygens. The latter can also result in hydrogen desorption and the formation of H_2_ gas.^39^ The oxidative healing process is the first step, which occurs in the absence of light and is followed by an aggregation of the nanosheets. As a result of the healing of oxygen vacancies, the hydrophobicity of the nanosheets increases, leading to their aggregation in solution, as observed via SEM and DLS. In the event of nanoparticle aggregation, the LSPR peak exhibits a red-shift due to the overlapping of the electron cloud. Therefore, the second step of the hypsochromic absorbance decrease is a result of nanosheet aggregation.^40^

When considering the spatial organization of the nanosheets from the SEM analysis, we can conclude that prior to light exposure, the nanosheets are organized as “layered nanosheets” and exhibit an LSPR peak after the addition of ascorbic acid. The first exposure to light caused the breaking and dispersion of the nanosheets due to the formation of oxygen vacancies, resulting in an increase in the intensity of the LSPR peak. In the absence of light, the oxygen vacancies are healed, and the nanosheets begin to aggregate to a new spatial structure (interlaced). The two spatial organizations, layered and interlaced, do not exhibit the same LSPR peak, probably due to their different 3D organizations, which is the reason for the first irreversible change in the LSPR peak. However, from the following cycles, the system consists of only two spatial organizations: dispersed and interlaced. The dispersed nanosheets, which are formed due to the ESPT from HPTS to the nanosheets, are rich in oxygen vacancies and exhibit a hypsochromic broadening of the LSPR peak. With time, the oxygen vacancies heal and the nanosheets aggregate into the interlaced structure.

In our work, we created a system that exhibits both a dynamic redox reaction and a dynamic self-organization of nanosheets. By controlling the redox reaction, we have a degree of control over the oxygen vacancies of MoO_3–*x*_. While we targeted the proof of our ability to control this redox reaction by an external photoacid, this property can be important for improving the known hydrogen evolution reaction catalytic activity of MoO_3_ nanosheets.^30^ The intercalation of protons to MoO_3_ can also be used for charge storage in protonic batteries.^41^ Hence, our new mechanism for charging MoO_3_ by both protons (from the photoacid) and electrons (from ascorbic acid) upon light irradiation represents an interesting new avenue. Due to the importance of MoO_3_ nanostructures in a wide array of applications, our new introduction and understanding of a mechanism that can change their properties are important for new developments in this field.

## Supporting Information Available

Detailed experimental section and supporting UV–vis spectra and ζ potential measurements (PDF)
