# Supplementary material for: Light-Triggered Reversible Change in the Electronic Structure of MoO3 Nanosheets via an Excited-State Proton Transfer Mechanism
Source: Nano Lett. 2024 Jan 30;24(6):1936–43. doi: 10.1021/acs.nanolett.3c04209 (PMC10870760; doi:10.1021/acs.nanolett.3c04209)
Supplement: Supplementary file 1 — nl3c04209_si_001.pdf [file nl3c04209_si_001.pdf]

Supporting Information

Light-triggered reversible change in the electronic structure of MoO_3 nanosheets via an excited-state proton transfer mechanism

Yuval Gilad Barzilay, Anna Yucknovsky, and Nadav Amdursky*

Schulich Faculty of Chemistry, Technion – Israel Institute of Technology, Haifa 3200003, Israel.

*Corresponding author e-mail: amdursky@technion.ac.il

Experimental Section

Synthesis of Nanosheets: MoO_3 nanosheets were prepared by a liquid exfoliation methodology. 200 mL of acetonitrile was added to 200 mg of MoO_3 powder, which was manually ground using a mortar and pestle. This was followed by dispersing the product in 50 ml of H_2O:ethanol (1:1) solution. The mixture was sonicated in ice water for 2 hours, after which the mixture was centrifuged at 8000 RPM for 30 minutes. The supernatant was collected and stored at 4°C.

Experimental Setup: 5 mM HPTS solution was prepared in H_2O:ethanol (1:1). 300 µL of the HPTS solution and 300 µL of 0.5 M ascorbic acid solution to which was added 10 µL of NaOH 1 M, were added to 1 mL of the nanosheets solution. The mixture was further diluted with 1.4 mL of H_2O:ethanol (1:1) solution and gently shaken for 30 minutes prior to experiments. The light source used for the excitation of the photoacid was a 405 nm LED, 3 mm in diameter, 540 mW (ThorLabs). For Absorption experiments, the light source was adjacent to the cuvette inside the UV-Vis spectrophotometer, and control over the light was done remotely, with the array of light emitted illuminating most of the sample. For DLS experiments, the light source was adjacent to the cuvette outside of the Zeta sizer illuminating the whole sample (the sample volume was smaller (700 µL) than the volume used for UV-Vis absorption measurements (1 mL). After the illumination period, the cuvette was immediately placed within the device to start the measurement. For SEM measurements, prior to the spin-coating process, the sample was illuminated for 5 minutes, with the same light source.

Spectroscopic Experiments: UV–Vis measurements were carried out on an Agilent Cary 60, fluorescence measurements were performed using a HORIBA Fluorolog fluorometer, and dynamic light scattering (DLS) measurements were carried out on a Malvern Instruments Zetasizer Nano. A quartz cuvette with a path length of 1 cm was used for both the UV–Vis and fluorescence measurements, and a plastic 1 cm was used for DLS.

X-ray Photoelectron Spectroscopy (XPS): XPS measurements were performed in an analysis chamber (UHV – 210-10 Torr during analysis) using a Versaprobe III – PHI Instrument (PHI, USA). The samples were irradiated with a focused X-ray AlKα monochromated X-ray source (1486.6 eV) using an X-ray beam of 200 microns, 50 W, 15 kV. The outcoming photoelectrons were directed to a spherical capacitor analyzer. The sample charging was compensated by a dual beam charge neutralization based on a combination of a traditional electron flood gun and a low-energy argon ion beam. The solutions were drop-cast on gold waivers and left to dry for

24 hours in vacuum conditions. Survey spectra were recorded with a pass energy of 224 eV, a step size of 0.4 V, and a Dwell time of 20 ms, from which the surface chemical composition was determined. The core-level binding energies of the different peaks were normalized by setting the binding energy for C1s at 284.8.0 eV.

Scanning Electron Microscopy: The spatial organization of the nanosheets was analyzed with Zeiss Ultra-Plus FEG-SEM. Samples were spin-coated on silica wafers, 2000 RPM for 2 minutes. The samples were left in vacuum conditions overnight before imaging.

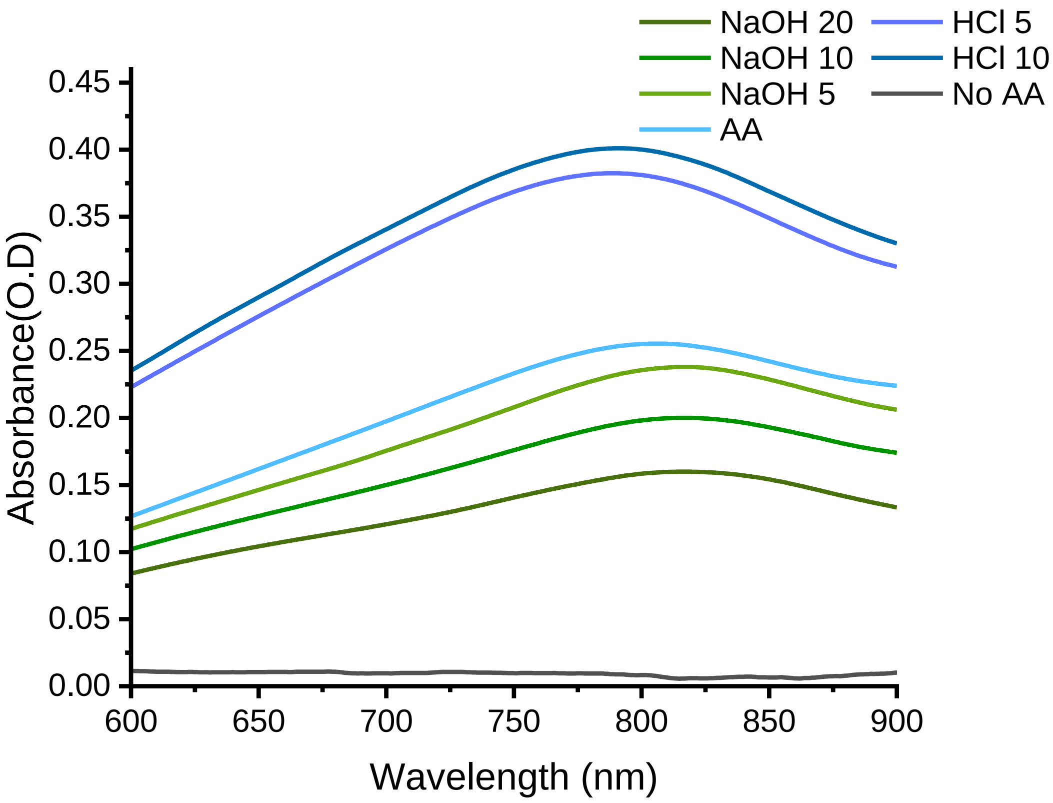

Figure S1. The addition of ascorbic acid (AA) to the MoO_3 solution results in the appearance of an LSPR peak in the Vis-IR region. Titration of MoO_3 nanosheets with ascorbic acid, with different amounts of HCl or NaOH (in µL). When the concentration of NaOH increases, the intensity of the LSPR peak decreases, and a bathochromic shift occurs, when compared to the solution with only ascorbic acid. The opposite effect occurs with the addition of HCl.

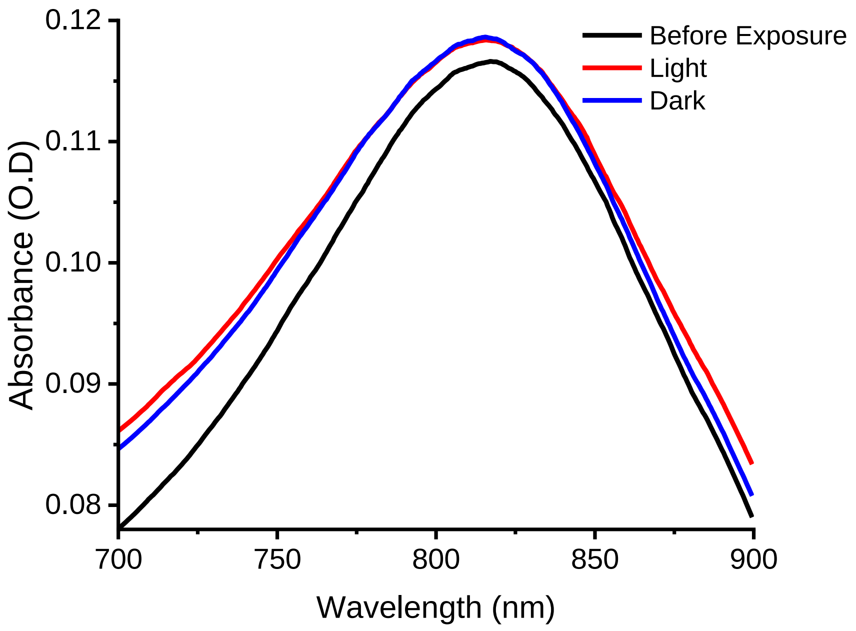

Figure S2. The first exposure to 405nm light, for 5 minutes, causes an irreversible rise of the LSPR peak.

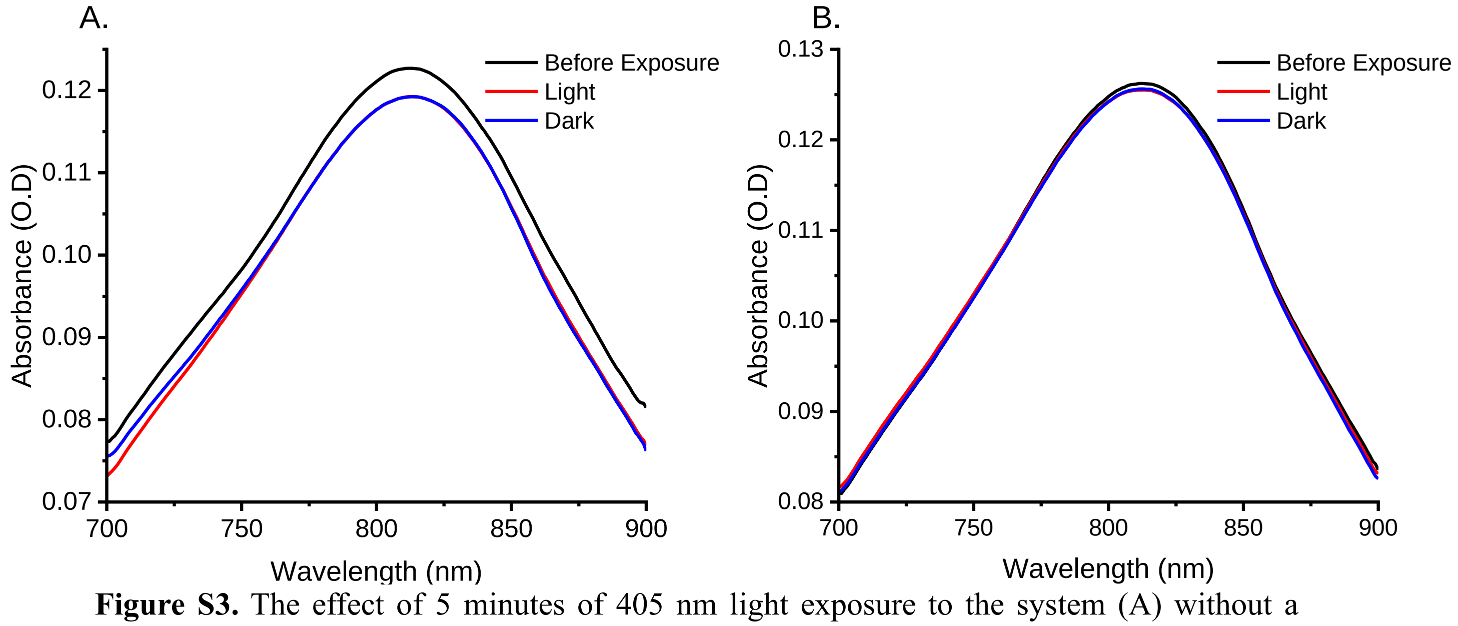

Figure S3. The effect of 5 minutes of 405 nm light exposure to the system (A) without a photoacid and (B) with MPTS instead of HPTS. In the absence of a photoacid, the exposure to light caused an irreversible decrease in the LSPR peak. Exposure to light in the presence of MPTS caused a minor irreversible decrease in the LSPR peak.

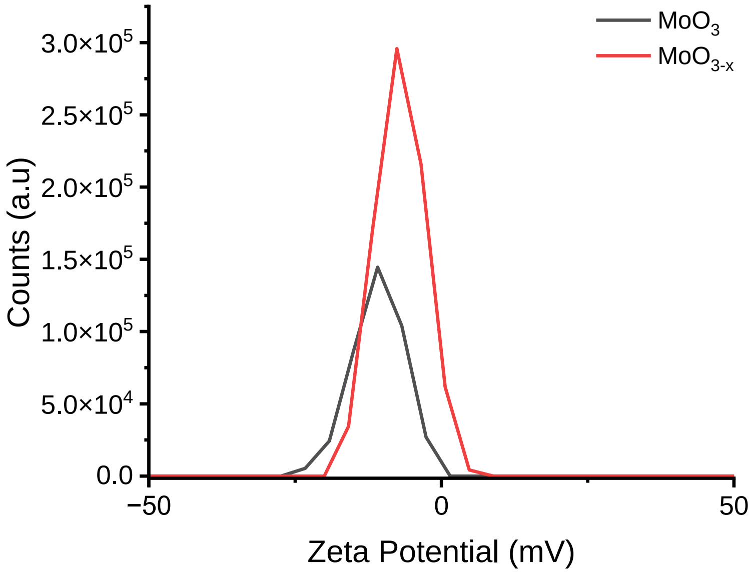

Figure S4. Zeta Potential of MoO_3 nanosheets and MoO_{3-x} nanosheets. As seen from the results the reduction reaction does not change the zeta potential of the nanosheets.
